# The Use of PCB Scrap in the Reduction in Metallurgical Copper Slags

**DOI:** 10.3390/ma16020625

**Published:** 2023-01-09

**Authors:** Albert Smalcerz, Tomasz Matula, Michal Slusorz, Julia Wojtasik, Weronika Chaberska, Szymon Kluska, Lukasz Kortyka, Lukasz Mycka, Leszek Blacha, Jerzy Labaj

**Affiliations:** 1 Department of Industrial Informatics, Faculty of Materials Science, Silesian University of Technology, Krasinskiego 8, 40-019 Katowice, Poland; 2 Department of Metallurgy and Recycling, Faculty of Materials Science, Silesian University of Technology, Krasinskiego 8, 40-019 Katowice, Poland; 3 Faculty of Materials Science, Silesian University of Technology, Krasinskiego 8, 40-019 Katowice, Poland; 4 Łukasiewicz Research Network—Institute of Non-Ferrous Metals, Sowińskiego 5, 44-100 Gliwice, Poland

**Keywords:** printed circuits board, scrap, metallurgical slag, reduction

## Abstract

The article presents the results of a study on metallurgical sludge reduction using electronic waste such as Printed Circuit Boards (PCBs). Two aspects were taken into account when selecting such a reducer, namely the environmental aspect and the technological aspect. The research was an attempt to use waste metal-bearing material of which the effective management causes many problems from an environmental point of view. In the technological aspect, the specific chemical composition of this waste was taken into account. Its gasification yields significant amounts of hydrocarbons, which are excellent reducing agents in such process. The separation of these compounds may additionally cause the mixing of the molten slag, which should result in faster separation of the formed metal droplets and the molten slag. In the case of the fragmented PCB (Printed Circuit Board) reducer used in this study, a significant degree of copper removal was achieved, as much as 92%. As the reduction-process time increased, the degree of copper removal also increased. For the 1 h process, the average value of copper removal was 60%, and for the 4.5 h process it was over 70%. The case was the same with the addition of reductant: as the amount of reductant added to the process increased, an increase in copper removal was observed. With the addition of 30 g of the reducing agent (per 65 g of slag), the degree of copper removal was over 90%.

## 1. Introduction

PCBs (Printed Circuit Board) are the basic component commonly used in the production of various types of electronic products. The development of technology in the field of electronic products’ production, with the simultaneous growing requirements as to their quality, resulted in a significant increase in the number of printed circuit boards in the market. At the same time, unfortunately, the lifetime of many electronic products has decreased, mainly due to their rapid functional aging. It is estimated that approx. 3% of the so-called PCBs constitute electronic scrap WEEE (Waste of Electrical and Electronic Equipment) [1]. The PCB consists of three layers, i.e., a non-conductive substrate or a laminate, metallic conductive paths as well as other additional elements mounted on the board. In terms of materials, PCBs mainly contain metals, ceramics and plastics with an admixture of flame retardants. The content of the individual material groups is diverse and largely depends on the technology and type of products. Table 1 summarizes the estimated content of the individual components in PCBs [2,3,4,5,6,7,8,9,10,11,12,13].

The PCB recycling and disposal technologies can be divided into two groups. The first one is based on pyrometallurgical, hydrometallurgical and pyrolysis processes [14,15,16,17,18,19,20,21,22,23,24,25,26,27,28,29,30,31,32,33,34,35,36,37]. The second group of technologies is based on mechanical operations such as crushing scrap and separation of the obtained fractions with subsequent chemical treatment. The basic problems related to the recycling and disposal of PCBs result from their complex structure and diverse material composition. The currently used PCB recycling technologies are focused mainly on the recovery of metals, for which printed circuits account for approximately 30% of the weight on average. The remaining waste consists of plastics and ceramics, which are not recyclable and end up in landfills.

Metallurgical slag-reduction processes are directed not only at recovering the metals they contain, but also at obtaining secondary waste slag that can be easily and safely recycled or landfilled/disposed of/or stored. Both solid and gaseous reducers are used in pyrometallurgical copper slag-reduction processes. The first group includes coal, coke, coke breeze, anthracite and graphite [38,39,40,41]. Research has also been carried out on the use of fine-grained coal-bearing waste materials as the reducing agents in the analyzed process, resulting from coal enrichment and processing [42,43,44]. The gaseous reducing agents used in the processes of copper recovery from slags are usually hydrogen, methane, carbon monoxide or natural gas [45,46,47]. It should also be mentioned that there are also studies aimed at replacing the solid carbon reducing agents with biomass and waste oils [48,49,50,51,52,53,54,55,56].

The main objective of this work was to determine the possibility of using PCB scrap as a reducer in the processing of copper slag. On the one hand, the recovery of valuable metals contained in the processed slag and PCB (copper, lead) was taken into account, and on the other hand, the possibility of obtaining secondary slag in the process, which could be directly used in other technologies, was also taken into account.

## 2. Materials and Methods

The copper slag from the industry was used for the reduction experiments. Table 2 presents the slag’s chemical composition.

The analysis of the microstructure was performed with scanning electron microscopy (SEM) on the Hitachi 3400N apparatus equipped with an X-ray spectrometer with Thermo Scientific NORAN EDS energy dispersion analyzer and Thermo Scientific System SIX (Electron Probe Microanalysis) microanalysis unit (Thermo Electron Scientific City, Madison, WI, USA). The morphology of the samples was imaged using the secondary electron detector (Secondary Electron—SE) and Environmental Secondary Electron Detector (ESED). The phase composition analysis was conducted by using the X-ray phase analysis method with an X-ray diffractometer (XRD) [43].

The research cycle consists of the following stages:

1. Preparation of PCB waste for the reduction process.
2. Conducting thermogravimetric tests of selected fractions of PCB waste.
3. Conducting reductive remelting of copper slags with the use of PCB waste reducer.

**Figure 1:**
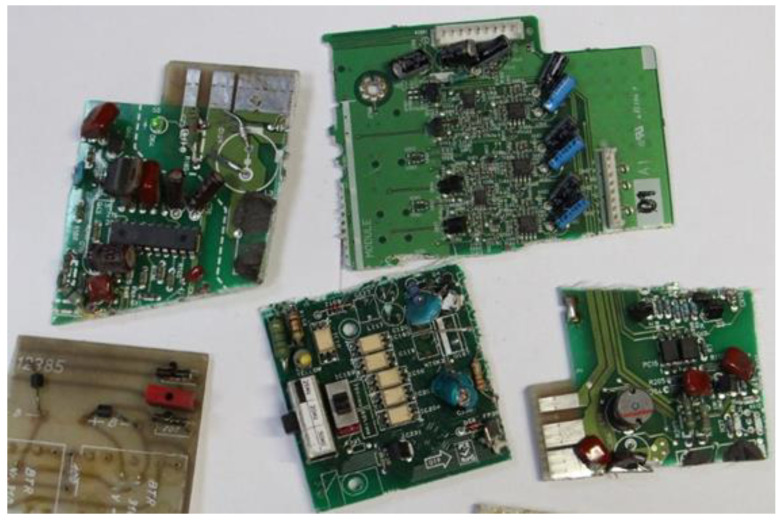
PCB scrap photo.

Due to the fact that the type of electronic scrap in the form of the tested PCBs was varied (Figure 1), it required appropriate preparation for further testing. This process included the following operations:

- Remove all capacitors from materials.
- Guillotine cutting of motherboards into fragments smaller than 4 × 4 cm.
- Grinding the obtained mainboard fragments on a Schröder grinder.
- Classification of the fragmented material into four material fractions with a grain size of: <1.6 mm, 1.6 ÷ 3.15 mm, 3.15 ÷ 6.3 mm, >6.3 mm, respectively.

**Figure 2:**
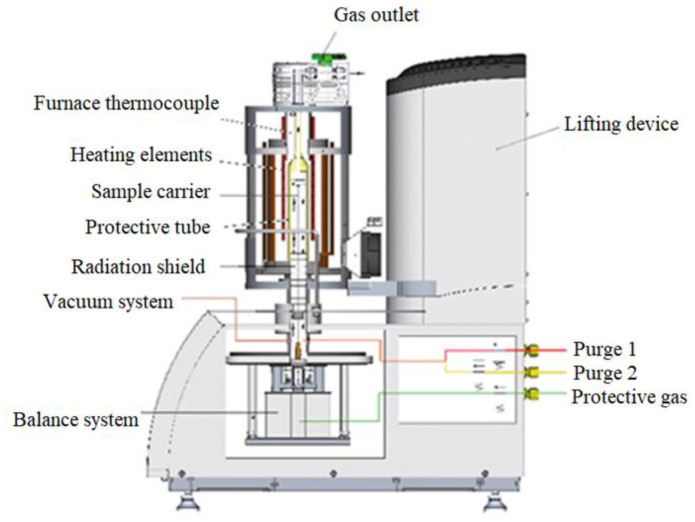
Diagram of the STA 449 F3 Jupiter thermal analyzer.

The basic device used in thermogravimetric studies was the thermal analyzer by Netzach, model STA 449 F3 Jupiter. The figure of this analyzer is shown in Figure 2.

The F3 Jupiter analyzer allows to carry out a number of measurements including thermal analysis. The carriers/heads included in the equipment of the used device allow for thermogravimetric measurements (TG) and differential thermal analysis (DTA) of the tested materials. The analyzer is equipped with a graphite furnace operating in a protective atmosphere (argon, helium), and enables measurements to be carried out at temperatures up to 2000 °C. During operation, the furnace is cooled intensively with water.

The PCB sample, located in the alundum crucible, was putted on the TG thermoanalyzer carrier. Then, after analyzer calibration, the appropriate gas was introduced into the analyzer chamber and it is flow rate was determined at a given level. The sample was heated to the temperature of 1300 °C, at a rate of 20 °C/min, and then isothermally heated at this temperature for 20 min. After this period, the sample was cooled to 800 °C. Crucibles with an internal diameter of 6 mm were used in the tests. The weight loss of the sample recorded in the experiment during its heating was assumed as the content of volatile parts in the tested material. Thermogravimetric tests were carried out for four PCB fractions with a grain size of <1.6 mm, 1.6 ÷ 3.15 mm, 3.15 ÷ 6.3 mm, >6.3 mm, respectively. The procedure was the same for all experiments.

**Figure 3:**
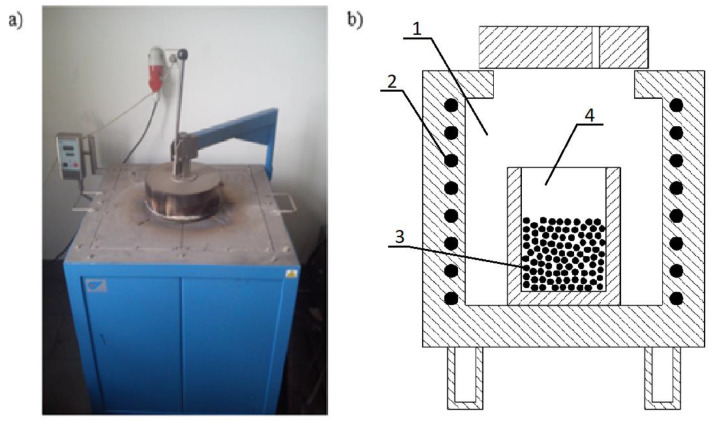
Resistance pit furnace: (**a**) general view, (**b**) diagram of the working chamber: 1—working chamber, 2—heating elements, 3—charge, 4—ceramic crucible.

The slag-reduction processes were carried out in a resistance pit furnace by Czylok, model PT 40/1300. The general view and the diagram of the resistance furnace working chamber is shown in Figure 3. The operating parameters of this unit are summarized in Table 3. The use of this type of furnace in laboratory studies allowed easy loading of the charge, as well as the possibility of controlling and observing the processes taking place in the crucible during the experiment.

**Figure 4:**
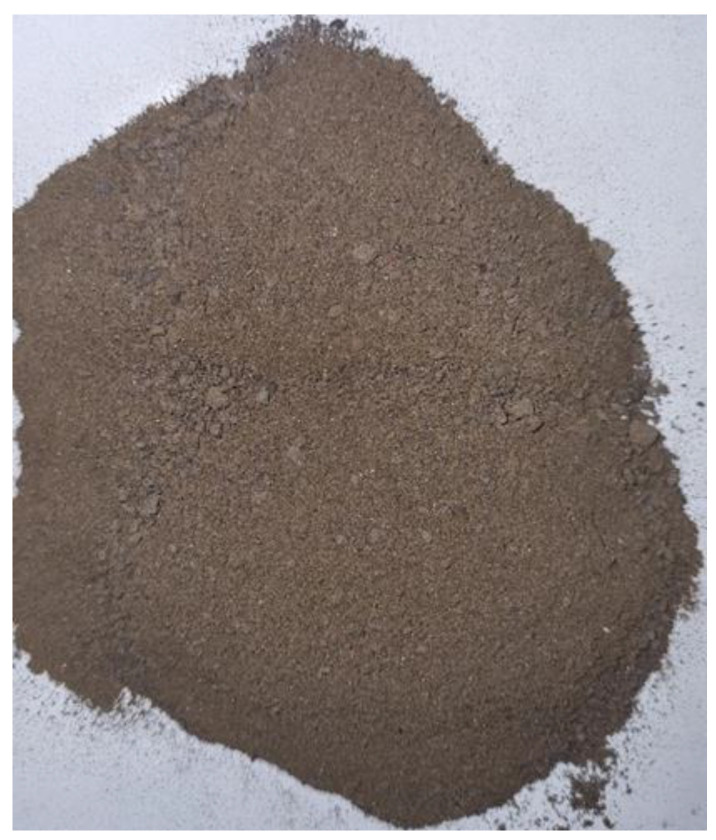
Photo of the slag used in the research.

The process was carried at a temperature of 1300 °C in alundum crucibles. This temperature was considered optimal on the basis of the preliminary tests results. The mass of the slag was 65 g (Figure 4). The variable parameters in the tests were the following:

- weight of the reducer 10, 15 and 30 g,
- duration of the reduction process 1, 2, 3 and 4.5 h,
- electronic scrap grain fraction.

After each experiment, the post-process slag was analyzed for Cu, Pb and Fe content. Before the chemical analysis, the slag sample was completely ground on a planetary mill whose grinding chamber was made of zirconium oxide (ZrO_2_). The final grain size of the sample was <0.2 mm, and it was the analytical sample. The mass of 2.0000 g of the slag prepared in this way (+/−0.0001 g) was transferred to a weighing vessel. The slag sample was then mixed with 2.0000 g (+/−0.0001 g) of a binder, which was anhydrous crystalline cellulose with purity p.a. In the next stage, the entire sample was pressed in a hydraulic press (Herzog, model HTP 40, Osnabrück, Germany). The pellet obtained in this way was analyzed for its elemental composition using a Primus II X-ray fluorescence spectrometer from Rigaku. Measurements were carried out using analytical programs used in the laboratory for quantitative and semi-quantitative SQX analyses.

To determine the chemical composition of the obtained metallic alloy, the Atomic Absorption Spectrometry (AAS) method was used to identify the copper, lead and iron content. Samples were taken from the metallic fraction in the shape of chips weighing 500 mg. This material was dissolved in concentrated nitric acid (V) and was heated to 60 °C. The next step was to supplement the solution with the addition of distilled water. The resulting solution was diluted with distilled water in a ratio of 1 to 99 (*v*/*v*). The chemical composition of the alloy was analyzed using a Solar M Series spectrometer from Unicam (Cambridge, England), obtaining the following characteristic analytical line lengths for the identified elements: Cu (λ = 324.8 nm); Pb (λ = 217.0 nm) and Fe (λ = 248.3 nm). The results obtained were expressed as mass percentages.

## 3. Research Results and Their Discussion

The slag used in the study was characterized by a fine-grained structure, where the particle size ranged from a few to tens of micrometers. Microstructure studies revealed a complex and heterogeneous structure, as well as a varied morphology characterized by particle shapes ranging from flat to spherical.

**Figure 5:**
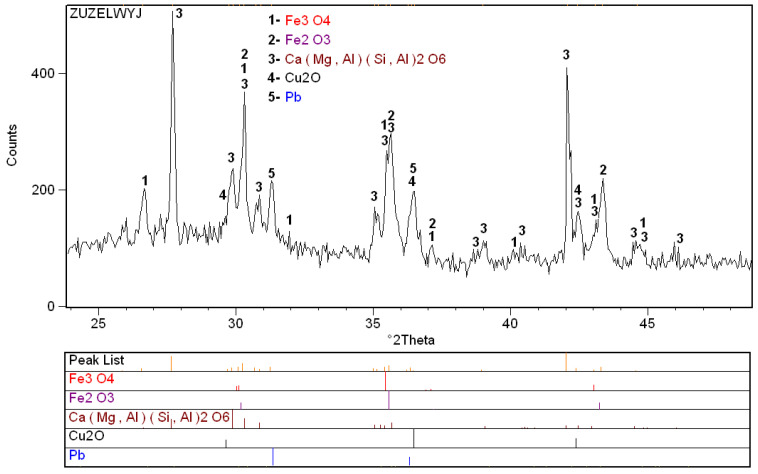
XRD of the initial slag [43].

Phase analysis of the slag using XRD X-ray diffraction revealed the presence of the following phases: Fe_3_O_4_, Fe_2_O_3_, Cu_2_O, Pb, Ca(Mg, Al)(Si, Al)_2_O_6_. Due to the complex nature of the diffraction lines for the obtained diffractograms, clear identification of the phases is difficult. Figure 5 shows an example of the diffraction line pattern for the copper slag used in the study.

The thermal decomposition of PCB was investigated using the thermogravimetric method.

**Figure 6:**
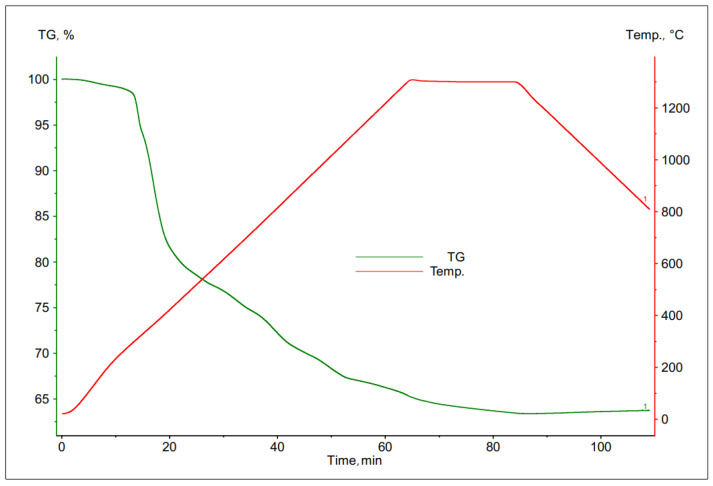
Change in mass of a sample with grain size <1.6 mm during its heating.

**Figure 7:**
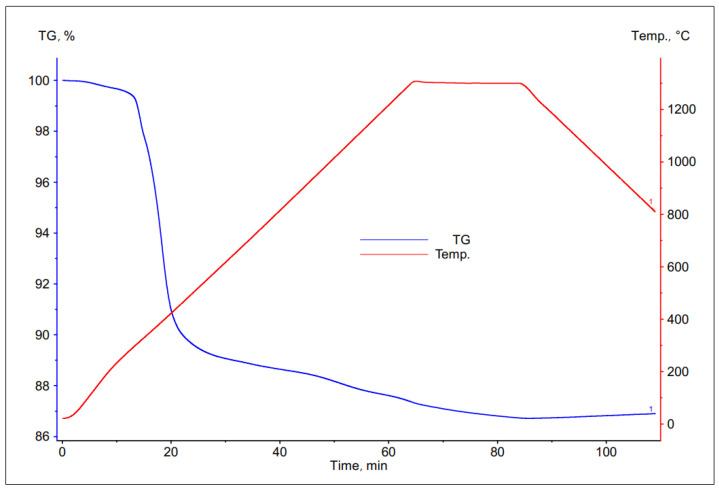
Change in mass of a sample with grain size 1.6 ÷ 3.15 mm during its heating.

**Figure 8:**
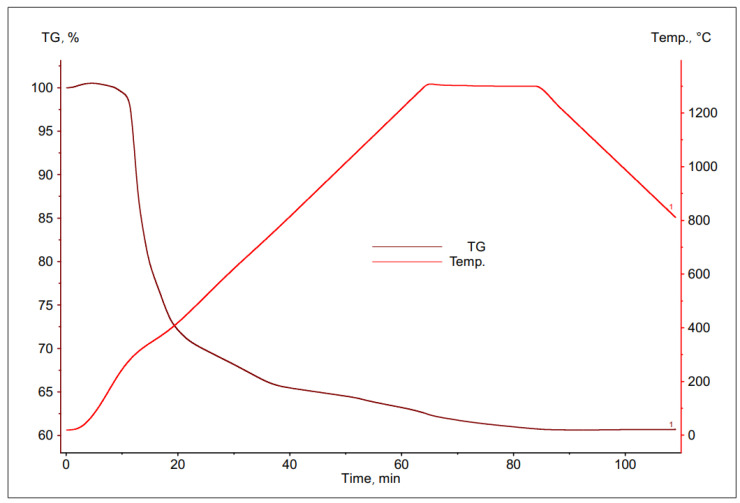
Change in mass of a sample with grain size 3.15 ÷ 6.3 mm during its heating.

**Figure 9:**
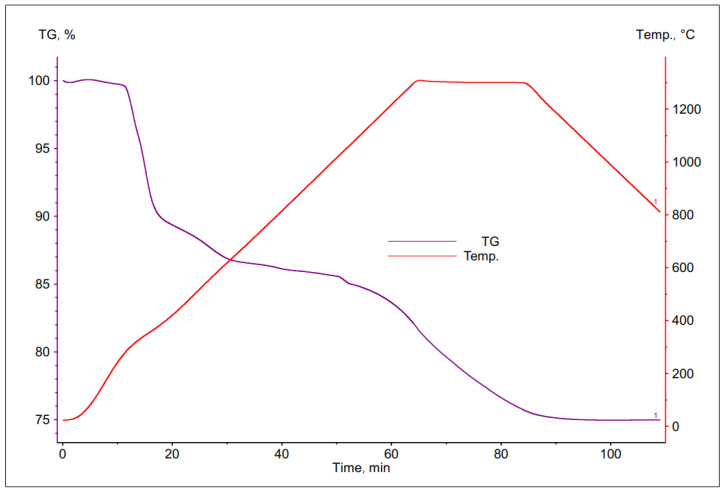
Mass in change of a sample with grain size > 6.3 mm during its heating.

As a result of the studies, a number of TG curves were obtained, which are shown in the Figure 6, Figure 7, Figure 8 and Figure 9. Comparing the following curves of weight loss of individual fractions of electronic materials as a function of time, significant differences were found in the nature of the curves and in the values of weight loss of the individual samples. The summary of the results of the TG curve analysis is presented in Table 4.

Based on the analysis of the obtained thermogravimetric curves, it was found that for most of the tested materials, a significant loss of weight was recorded, ranging from 25% to 39%. The only exception here was the sample number 2, for which the registered weight loss was about 13%.

In case of the data contained in Table 4, it can be concluded that the largest total weight loss was characteristic for samples No. 1 and 3, the grain size of which was <1.6 and 3.15 ÷ 6.3 mm, respectively. This may indicate a high volatile matter content in these samples as well as a low metallic content. Sample no. 4, with the largest grain size, was characterized by a slightly smaller loss of weight, about 25%. The smallest percentage weight loss was recorded for the sample with a grain size of 1.6 ÷ 3.15 mm, which may indicate that it had the highest metallic fraction content and is most likely to have the lowest ability to remove copper from the slag.

Comparing the thermogravimetric results of the PCB samples presented in the literature with the results of our tests, there are some differences. They mainly concern the values of the total mass loss of the samples. As noted earlier, the obtained mass losses in this work, for various fractions of PCB scrap, were in a relatively wide range from 13% to 37%. These differences may be caused by the type of resins found in the PCBs, as well as the content of the metallic parts in them. As shown in the work [57], for the samples with a low metal content (samples only with copper parts and soldering pads), the mass losses up to 75% were obtained, while for the samples with metallic parts, the max was 50%. In the work of Orton et al. [58], the results of the PCB samples heated with a rate equal to 5, 10 and 20 k min^−1^ are presented. The maximum rate of a sample distribution was observed at temperatures of 680–800 K. The maximum loss in the sample mass was 24%. The similar results are presented in [59]. The authors of this work were studying three types of printed circuit boards and obtained a mass loss of 26%. At the same time, they observed two DTG maxima at temperatures of 593 K and 629 K. The differences in the temperatures of these peaks were attributed to the presence of different resins (epoxy resin and polyester resin) in the composition of the PCB samples, but also to flame retardants. These agents are one of the components of the PCB. Importantly, the temperature range specified in this work corresponding to the largest loss of mass of samples was in the range from 573 to 673 K.

Copper in slags formed in various pyrometallurgical processes for obtaining this metal is found either in dissolved form or in the form of droplets. Its content covers a very wide range from 0.5 to 15 wt.%. Therefore, smelters try to reduce copper losses in slags by recovering copper from them. This is achieved by various methods. When selecting these methods, the economic aspect is mainly taken into account, i.e., it is necessary to meet the condition that the expenditure on copper recovery does not exceed its value. It should be remembered that waste slags may be stored if their copper content does not exceed 1%.

When discussing the investigated process of Cu recovery from slags, it should be noted that copper in the slag, as an ionic liquid, occurs as Cu^+^ cations, which are combined with silicate anions or in the form of Cu_2_O.

An increase in the viscosity of the slag was observed, in the course of the reduction process, which is believed to prevent the metal droplets from falling. The copper solvation in the slag can be written as [43]:

(Cu_2_O) slag => 2(Cu^+^) slag + (O_2_^−^) slag (1)

The releasing of free electrons participating in the reduction in ions plays a key role in the slag-reduction process:

(O_2_^−^) slag + C => CO + 2e^−^ (2)

4(O_2_^−^) slag + CH_4_ => CO_2_+ 2H_2_O + 8e^−^ (3)

7(O_2_^−^) slag + C_2_H_6_ => 2CO_2_ + 3H_2_O + 14e^−^ (4)

The negative charge transfer mechanism can be active throughout the slag volume, so the transport of electrons is relatively fast.

Chemical analysis of copper slags confirms the presence of various elements at different oxidation levels. In the case of the iron contained in the slag, it has been shown that it can occur at both the second and third oxidation levels. Metal ions present in the molten slag may play an important role in electron transfer [60,61].

(Cu^+^)_s_ + e^−^ => [Cu]_m_ (5)

(Pb^2+^)_s_ +2e^−^=> [Pb]_m_ (6)

2(Fe^3+^)_s_ +2e^−^ => 2(Fe^2+^)_s_ (7)

(Fe^2+^)_s_ +2e^−^ => [Fe]_m_ (8)

In the publication [62], a limiting content of Fe was estimated, which can be as high as 0.28%. The content of this element can intensify the reduction process of Cu and Pb, according to the following reactions:

(Cu_2_O)_s_ + [Fe]_m_ => (FeO)_s_ + 2[Cu]_m_ (9)

(PbO)_s_ + [Fe]_m_ => (FeO)_s_ + [Pb]_m_ (10)

As shown in the work [63], the ratio of iron ions in the second oxidation state to iron ions in the third oxidation state strongly depends on the partial pressure of oxygen and the alkalinity of the slag. In addition, the presence of silica in the slag can, with an increase in its content, reduce the Fe^3+^/Fe^2+^ values and thus minimize the effect on the reduction process of copper from the slag

Given that this type of slag has a structure composed of silicate chains, the presence of other metals such as Na or Ca will affect the breaking of bonds between silicon and oxygen. Alkali elements play a special role here, whose ions will cleave silicate chains. The presence of additives such as CaO, MgO or Al_2_O_3_ in the liquid slag will reduce the solubility of copper. The occurrence of Cu^+^ and Fe^2+^, Fe^3+^ ions in the slag structure [64] is in the oxide form.

The reduction in the copper present in the slag with the carbon reducing agents goes via the following reactions:

Cu_2_O + C => 2Cu + CO (11)

2Cu_2_O + C => 4Cu + CO_2_ (12)

Cu_2_O + CO => 2Cu + CO_2_ (13)

**Figure 10:**
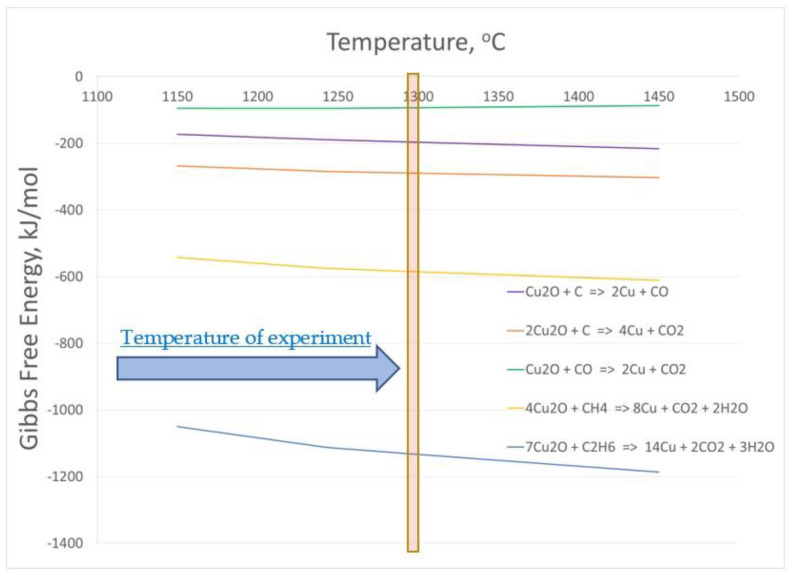
Gibbs free energy change for selected Cu_2_O reduction reactions.

The PCB waste material used in this study undergoes a degassing process during thermal treatment, during which significant amounts of hydrocarbons are produced. In order to determine the possibility of the course of the reduction reaction of copper oxides contained in the slag using hydrocarbons accumulated in the PCB material, changes in Gibbs free energy were analyzed for the course of the reaction in the temperature range of 1150 ÷ 1450 °C. The results of the analysis are shown in Figure 10. The formulas presented were obtained from the HSC database [58]. Based on the presented results, it can be concluded that from a thermodynamic point of view, all the studied reduction reactions can proceed in the analyzed temperature range.

Metal oxides with lower chemical affinity for oxygen are reduced first. In this case, Cu will undergo reduction first, followed by Pb and Fe. For copper, which is not bound to silica in silicate chains, it is very easy to reduce its content in slag to about 1%. Further reduction in the Cu content in slag occurs very slowly, and in this case the phenomenon of replacing copper ions with calcium ions is used.

In the process of reducing copper slags, the product is a metallic alloy, which remains in the form of a suspension of metal fines. Below a size of about 10 µm, these particles remain suspended on the surface of the liquid slag [65,66,67,68]. The course of the reduction process is particularly intense at its initial stage. In the industrial process at the stage during the pouring of liquid slag into the furnace working chamber, it is ca. 80% of Cu. This suggests that the reduction reactions occur as a result of the intensive mixing of slag and the reductant components. The rate of movement of the reduced metal droplets depends on several factors, including the mechanism associated with Brownian motion or as a result of turbulent motion [69].

As Czernecki’s research has shown, the process of removing copper from slag requires ensuring conditions in which rapid separation of the metallic phase from the slag phase is possible. This process is faster when we deal with mixing of the molten slag phase. It is possible, inter alia, by the addition of CaCO_3_, which decomposes with the release of CO_2_ and allows the molten slag to move. Considering what was mentioned above, the proposition of PCB scrap as a reducer for the slag copper removal process brings a beneficial element influencing its efficiency. This is due to the fact that under the influence of temperature, this material releases components in the form of numerous hydrocarbons, which causes slag mixing and at the same time the hydrocarbons take part in the reduction processes. This solution is a new approach to the analyzed slag removal process.

Table 5 summarizes the exemplary results of the copper slag reduction with the use of PCB as a reducer. Apart from the basic data concerning the experiments, this table also includes the mass of the melted alloy and second slag as well as the content of the metals in the slag and in the metallic alloys after the reduction process.

For each experiment, the relative degree of copper removal of the slag was additionally determined. The value of this indicator was estimated on the basis of the equation:

(14) $$S_{Cu}=\frac{\left(C_{Cu}^{0}-C_{Cu}^{k}\right)}{C_{Cu}^{0}}\times 100\%$$

where

$C_{Cu}^{0}\;\mathrm{and}\;C_{Cu}^{k}$—the initial copper content in the slag and the copper content in the slag, after the slag reduction process, respectively.

The collective results of the slag reduction are presented in Table 5.

**Figure 11:**
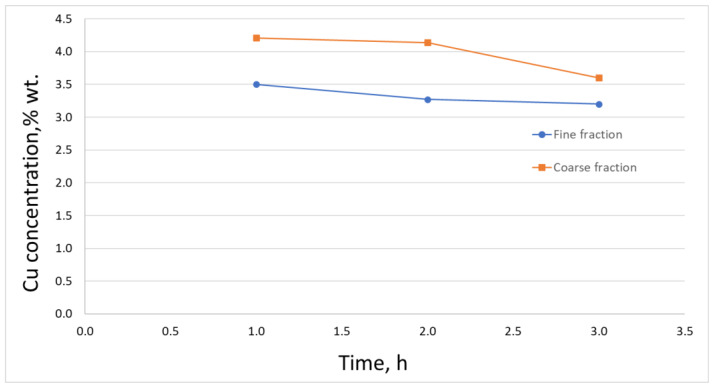
Copper content in the slag after the reduction process.

Analyzing the obtained results in terms of the change in the composition of the initial slag, a significant degree of copper separation was found. Along with the increase in the duration of the reduction process, the degree of copper removal increased. Hence, for the process lasting 1 h, the average degree of reduction was above 70%, and for the process lasting 3 h, it was over 85% (Figure 11).

**Figure 12:**
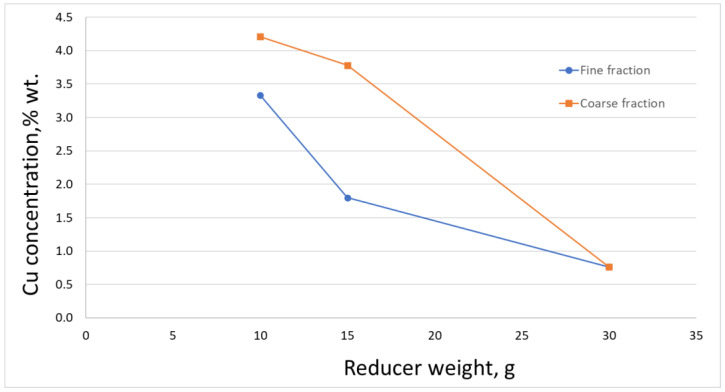
Copper content in the slag after the reduction process with different amounts of the reductant added.

Along with the increase in the amount of added reductant, an increase in the degree of copper removal was observed. With the addition of the reducing agent in the amount of 30 g (for 65 g of slag), the degree of copper separation was over 90% (Figure 12). The conducted research did not show a significant influence of the used grain size of the scrap (reducing agent) on the degree of copper removal from the initial slag.

In the reduction processes, from 5 g to over 20 g of Cu-Pb alloy was obtained. The main components of this alloy were copper (from 87% to 96% wt.%) and lead (from 3% to 9% by wt.%). The amount of the formed alloy increased with the increase in the duration of the reduction process and with the increase in the amount of the reducing agent added. In the first case, it is due to the increase in the amount of copper produced as a result of the reduction process, and in the second case, to the increase in the number of metals introduced into the process in the form of PCB scrap. On the other hand, the slag-reduction studies showed a slight degree of iron transition to the metallic alloy.

## 4. Conclusions

The research conducted in this work was aimed to determine the possibility of effective use of PCB waste in the reduction in copper slags. When proposing PCB scrap, its specific chemical composition was taken into account, i.e., a significant content of volatile components, mainly hydrocarbons. For the reducer used in the tests in the form of shredded electronic scrap (with the assumed experimental parameters), the degree of copper removal from the slag varied from 71% to 97%. As the duration of the reduction process increased, the degree of copper removal from the slag increased. This was also observed with the increase in the amount of the reducing agent added. It was also shown that the fractions with a high content of volatiles are better reducing agents than the fractions with a lower content of volatiles. At the same time, the final slag after reduction can be used as a technological additive for processing in a shaft furnace for the production of copper matte.

## Figures and Tables

**Table 1:** PCB material composition [2,3,4,5,6,7,8,9,10,11,12,13].

Type of Material		Min. Content, wt.%	Max. Content, wt.%	Average Content, wt.%
Metals	Cu	10	26.8	18.4
	Al	1.33	7	4.165
	Pb	0.60	4.2	2.4
	Zn	0.16	2.17	1.165
	Ni	0.11	2.35	1.23
	Fe	0.22	8	4.11
	Sn	1	6.48	3.74
	Sb	0.06	1.97	1.015
	Ag	0.011	0.363	0.187
	Sr	0.65	0.65	0.65
	Ta	0.07	0.07	0.07
	Au	0.0076	0.1	0.0538
	Ca	9.96	9.96	9.96
	Fe	2.08	2.08	2.08
	Cr	0.003	0.356	0.1795
	Mo	0.016	0.016	0.016
	Mn	0.05	0.05	0.05
	Pd	0.001	0.0294	0.0152
	Br	4.94	6.5	5.72
Ceramics	SiO_2_	11.3	41.86	26.58
	Al_2_O_3_	6	9.35	7.675
	CaO	1.9	6.7	4.3
	MgO	0.081	0.22	0.1505
	BaO	0.0022	0.16	0.0811
Plastics	-	30	32.14	31.07

**Table 2:** The chemical composition of the copper slags.

Slag Component Content, wt.%						
Cu	Fe	Pb	SiO_2_	Al_2_O_3_	MgO	CaO
10.3	11.1	2.25	34.5	8.2	3.99	14.1

**Table 3:** Working parameters of a resistance pit furnace.

Work temperature	to 1350 °C
Work atmosphere	Air
Rated power	13.5 kW
Rated current	19.57 A

**Table 4:** Recorded weight losses of individual samples.

Sample Number	Sample Grain Size, mm	Loss of Weight, %
1	<1.6	36.60
2	1.6 ÷ 3.15	13.28
3	3.15 ÷ 6.3	39.36
4	>6.3	25.03

**Table 5:** Results of slag copper removal experiments.

No.	Charge Components			Reduction Time, h	Alloy Weight, g	Chemical Composition of Waste Slag, wt.%		
	Slag, g	Electronics				Cu	Pb	Fe
		Mass, g	Fraction, mm					
1	65	10	>6.3	1	5.45	1.36	1.45	10.28
2		10		2	5.47	1.11	2.22	10.99
3		10		3	6.67	1.82	2.2	10.89
4		15		3	7.64	2.08	2.08	10.76
5		30		3	17.57	0.6	1.61	11.21
6		10	3.15 ÷ 6.3	1	5.02	2.13	1.92	9.87
7		10		2	3.96	1.81	2.19	11.07
8		10		3	6.67	1.77	2.11	10.91
9		15		3	10.75	0.61	0.84	10.96
10		30		3	16.52	0.31	0.19	9.67
11		10	1.6 ÷ 3.15	1	5.45	1.19	1.4	8.66
12		10		2	4.27	1.42	1.6	9.75
13		10		3	4.58	1.91	2.08	9.94
14		15		3	11.98	0.71	1.08	9.89
15		30		3	18.84	0.51	1.49	10.38
16		10	<1.6	1	2.53	2.97	2.54	11.42
17		10		2	4.4	1.94	1.84	8.87
18		10		3	3.99	1.92	1.91	10.26
19		15		3	10.82	0.69	1.13	9.7
20		30		3	20.55	0.73	1.26	11.54

## Data Availability

Data sharing is not applicable to this article.
